# Diagnosis, Management, and Treatment of Vernal Keratoconjunctivitis in Asia: Recommendations From the Management of Vernal Keratoconjunctivitis in Asia Expert Working Group

**DOI:** 10.3389/fmed.2022.882240

**Published:** 2022-08-01

**Authors:** Jodhbir S. Mehta, Wei-Li Chen, Arthur C. K. Cheng, Le Xuan Cung, Ivo J. Dualan, Ramesh Kekunnaya, Nurliza Khaliddin, Tae-Im Kim, Douglas K. Lam, Seo Wei Leo, Florence Manurung, Nattaporn Tesavibul, Dominique Bremond-Gignac

**Affiliations:** ^1^Corneal & External Eye Disease Department, Singapore National Eye Centre, Singapore, Singapore; ^2^Department of Ophthalmology and Advanced Ocular Surface and Corneal Nerve Research Center, National Taiwan University Hospital, Taipei, Taiwan; ^3^Department of Ophthalmology, Hong Kong Sanatorium & Hospital, Hong Kong, Hong Kong SAR, China; ^4^Vietnam National Eye Hospital, Hanoi, Vietnam; ^5^Department of Ophthalmology and Visual Sciences, University of the Philippines, Quezon City, Philippines; ^6^Child Sight Institute, Jasti V Ramanamma Children’s Eye Care Center, LV Prasad Eye Institute, Hyderabad, India; ^7^Department of Ophthalmology, University Malaya Eye Research Center, Universiti Malaya, Kuala Lumpur, Malaysia; ^8^Department of Ophthalmology, Institute of Vision Research, Yonsei University College of Medicine, Seoul, South Korea; ^9^The Hong Kong Ophthalmic Associates, Hong Kong, Hong Kong SAR, China; ^10^Dr Leo Adult & Paediatric Eye Specialist Pte Ltd; Mount Elizabeth Hospital, Singapore, Singapore; ^11^Jakarta Eye Center Hospitals and Clinics, Jakarta, Indonesia; ^12^Department of Ophthalmology, Faculty of Medicine, Siriraj Hospital, Mahidol University, Bangkok, Thailand; ^13^Department of Ophthalmology, University Hospital Necker-Enfants Malades, APHP, OPHTARA, Paris, France; INSERM Unit UMRS1138, Team 17, Paris University, Paris, France

**Keywords:** vernal keratoconjunctivitis (VKC), ocular surface, ocular allergy, cyclosporine A (CsA), corticosteroids, MOVIA, consensus, recommendations (guidelines)

## Abstract

Vernal keratoconjunctivitis (VKC) is an underdiagnosed and underrecognized ocular surface disease with limited epidemiological data in Asia. It is more prevalent in warm, dry, and windy climates, and often has a substantial impact on a patient’s quality of life. In rare cases, VKC can be associated with vision loss, either through corticosteroid overuse or inadequate treatment of persistent inflammation. As a potentially severe and complex disease, there is variability with how VKC is managed across Asia and among the various allergic eye diseases. Diagnosis and treatment of patients with VKC is a challenge for many ophthalmologists, since no precise diagnostic criteria have been established, the pathogenesis of the disease is unclear, and anti-allergic treatments are often ineffective in patients with moderate or severe disease. In addition, the choice of treatment and management strategies used for patients varies greatly from country to country and physician to physician. This may be because of a lack of well-defined, standardized guidelines. In response, the Management of Vernal Keratoconjunctivitis in Asia (MOVIA) Expert Working Group (13 experts) completed a consensus program to evaluate, review, and develop best-practice recommendations for the assessment, diagnosis, and management of VKC in Asia. The expert-led recommendations are summarized in this article and based on the currently available evidence alongside the clinical expertise of ophthalmologists from across Asia with specialism and interest in the ocular surface, VKC, and pediatric ophthalmology.

## Introduction

**FIGURE 1:**
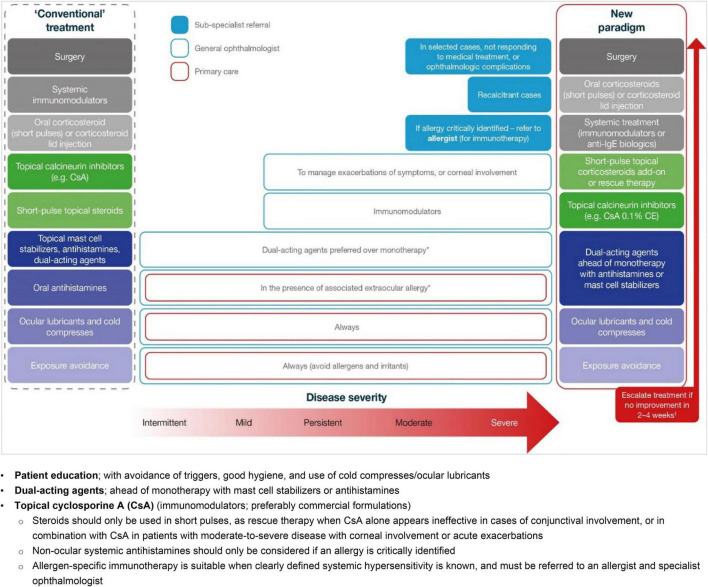
A new paradigm for the stepwise management of VKC based on disease severity. Adapted from Leonardi et al. (28). *In the case of associated rhinitis, consider treatment according to Allergic Rhinitis and its Impact on Asthma (ARIA) protocol; ^†^No improvement is defined as no improvement in symptoms or changes in conjunctival, papillary or ocular surface clinical signs. CE, cationic emulsion; CsA, cyclosporine A; IgE, immunoglobulin E.

The key findings and best-practice recommendations of the MOVIA Expert Working Group, as shown in Table 1 (with a summary of a stepwise management approach according to disease severity shown in Figure 1), are based on the available literature, evidence, and clinical experience at the time of writing. All relevant information and literature pertaining to the discussions exchanged in the consensus program are detailed hereafter. These expert-led, best-practice recommendations are not intended as clinical practice guidelines.

**TABLE 1:** Expert-led recommendations from the MOVIA Expert Working Group.

**Assessment and diagnosis**
• Slit-lamp examination and eversion of eyelids.
• Ocular surface staining, tear film stability, and breakup pattern; if available.
• Adjunctive tests should be considered in the context of a multidisciplinary team approach, if required and if locally available.
**Management should follow a stepwise approach with active patient education**
• Address triggers and aggravating factors (e.g., environment and allergens).
• Maintain ocular health, including frequent hand, face, and hair washing.
• Use of ocular lubricants and cold compresses should always be considered.
**Several treatments are effective for reducing the symptoms of VKC** *(For the use of any eye drops on the ocular surface, it is recommended to use preservative-free compounds, where possible, to minimize ocular surface toxicity)*
**Conventional topical anti-allergic drugs**
° Dual-acting agents, antihistamines, and mast cell stabilizers are all effective for reducing signs and symptoms of mild or moderate VKC.
° Dual-acting agents should be considered ahead of monotherapy with antihistamines or mast cell stabilizers.
**Cyclosporine CsA 0.1% cationic emulsion (CsA 0.1% CE)**
° Topical CsA 0.1% CE should be considered for patients with in moderate-to-severe or persistent VKC.
° Patients should be instructed on how to apply CsA eye drops to minimize stinging or burning on instillation, such as using artificial tears prior to instillation.
**Topical corticosteroid eye drops**
° In patients with only conjunctival involvement, topical corticosteroids should be reserved for use after loss of control or persistence of symptoms with immunomodulators (such as CsA).
° Topical corticosteroids are effective for the management of acute exacerbations, or when the cornea is involved, and preferably only introduced in patients with more severe disease. In these individuals, corticosteroids should be used in combination with CsA to account for the fact that CsA may require ≥1 week to act.
° Because of an increased risk of adverse events/or vision loss with chronic use, topical corticosteroid eye drops should be used in short pulses (alone or in combination with CsA) under the supervision of an ophthalmologist and tapered rapidly.
**Tacrolimus**
° In regions where available, tacrolimus should be reserved for patients with severe VKC that is refractory to CsA. It can be considered as a treatment for moderate-to-severe VKC in patients with allergy of the eyelid, but please note this may be off-label.
**Vasoconstrictors**
° Vasoconstrictors are not recommended for the treatment of VKC.
° If used to address hyperemia, vasoconstrictors should be used with caution and only for a short period due to adverse events.
**Non-steroidal anti-inflammatory drugs (NSAIDs)**
° NSAIDs are not recommended as they do not target the specific inflammatory mechanism associated with VKC.
**Systemic antihistamines**
° Second-generation systemic antihistamines are preferred over older first-generation antihistamines.
**Allergen-specific immunotherapy**
° Allergen-specific immunotherapy is only recommended when clearly defined systemic hypersensitivity to an identified allergen exists.
° Patients requiring allergen-specific immunotherapy should be referred to an allergist or specialist ophthalmologist.
**In selected patients with ocular complications or persisting symptoms following prior treatments**
° Surgery, oral corticosteroids (short pulses) or corticosteroid lid injection, or systemic treatment with immunomodulators or biologics may be appropriate options for use by corneal specialists.

### Background

#### Definition

Vernal keratoconjunctivitis (VKC) is a recurrent form of ocular allergy characterized by severe and often bilateral chronic inflammation of the ocular surface, which can result in permanent injury or visual disabilities if not adequately recognized and treated. VKC is a T-lymphocyte-mediated disease with subsequent immunoglobulin (Ig)E-mediated chronic inflammation involving eosinophils, mast cells, lymphocytes, and structural cell activation (1–4).

#### Epidemiology

Allergic conjunctivitis alone has an estimated worldwide prevalence of 6–30% in the general population and is estimated to occur in up to 30% of children, either alone or in association with allergic rhinitis (5). VKC is an underdiagnosed and underrecognized ocular surface disease and epidemiological data are limited. VKC is most frequent in Asia, Central and West Africa, and South America, but is also common in the Mediterranean area, North America, and Australia (1, 6–9). A recent epidemiological survey has provided insight into the frequency of VKC across Europe, where a prevalence of <1–3.2/10,000 overall and 0.8/10,000 with corneal complications has been reported (9). In Japan, a prevalence of 1.2% has been described, and in African countries prevalence ranges from 4.0 to 11.1% (8). However, the prevalence of VKC in much of Asia is currently unknown and current estimates may be inaccurate (6)—this is because the clinical form is generally mild and self-limiting, and access to care varies significantly among Asian countries, meaning that many patients do not present to a clinic. In addition, many ophthalmologists categorize VKC as an ocular allergy, and therefore estimates can be misleading and underrepresent the true prevalence of the condition. Depending on geography and climate, the prevalence of VKC can vary widely across Asia, including within countries and regions. There remains an unmet need for ophthalmologists to accurately diagnose VKC and for precise estimates of prevalence, including differences in clinical presentation, across Asia.

VKC occurs mainly in children and young adults, with onset often occurring in the first decade of life (predominantly 5–10 years) (9). While it is considered a long-term disease with an average duration of 4–8 years (9), VKC generally subsides before or just after puberty, but can leave permanent lesions in patients with severe disease (7). The disease is more common among males than females, with a ratio of 3:1, but this difference may become less at older ages of onset (7, 10).

Environmental factors play an important role in the development of VKC, with increased frequency in warm, dry, and windy climates, and worsening severity as a result of air pollution and Asian dust storms (1, 7, 11, 12). The MOVIA Expert Working Group previously recognized that despite a typically seasonal trend, perennial forms of VKC are more widely reported (50–100% of the VKC population across different regions of Asia) with acute exacerbations in the spring–summer period; seasonal forms are more commonly reported in Hong Kong (up to 50% of patients with VKC) and to a lesser extent in South Korea (20%). The type of allergen responsible for aggravating VKC may explain its seasonal or perennial nature. For example, pollen may be responsible for seasonal VKC, while dust mites (or other indoor allergens) could be responsible for the perennial form.

#### Pathophysiology

VKC is a T-helper-2 (Th2) lymphocyte-driven disease characterized by infiltration of the conjunctiva by a number of inflammatory cell types, including eosinophils, mast cells, and T lymphocytes (3, 4). This differs from atopic keratoconjunctivitis (AKC), which has been shown to involve both Th1 and Th2 inflammatory cascades (13). Increased levels of tumor necrosis factor (TNF) alpha, histamine, tryptase, IgE, and IgG antibodies are observed on pathologic examination of tears (14). It is believed that the exaggerated IgE response observed with VKC in response to common allergens may be a secondary event (4). Mast cells and basophils cause the immediate reaction (through the release of histamine) and the recruitment of inflammatory cells (lymphocytes and eosinophils). This results in the release of a number of pro-inflammatory cytokines, including (but not limited to) interleukin (IL)-4, IL-5, and IL-13, as well as other toxic cell mediators (such as eosinophil cationic protein, eosinophil-derived neurotoxin/eosinophil protein X [EDN/EPX]) that result in corneal damage (4, 15). Release of these factors mediates the remodeling, ocular inflammation, and itch that are commonly associated with VKC.

Management strategies should continue to be focused on tackling specific aspects of the clinical presentation and pathophysiology of VKC.

#### Clinical Forms

**FIGURE 2:**
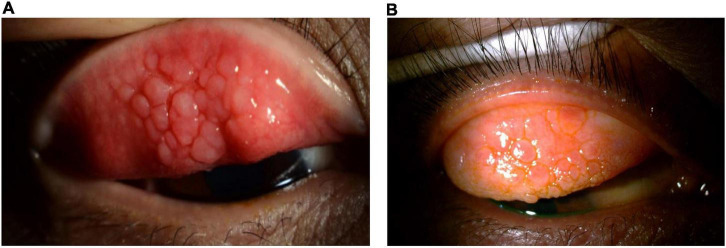
Clinical subtypes of VKC: *Tarsal form*. The tarsal form is characterized by large, cobblestone-like papillae on the upper tarsal conjunctiva. (A) Image courtesy of Jodhbir S. Mehta; (B) image courtesy of Douglas K. Lam.

**FIGURE 3:**
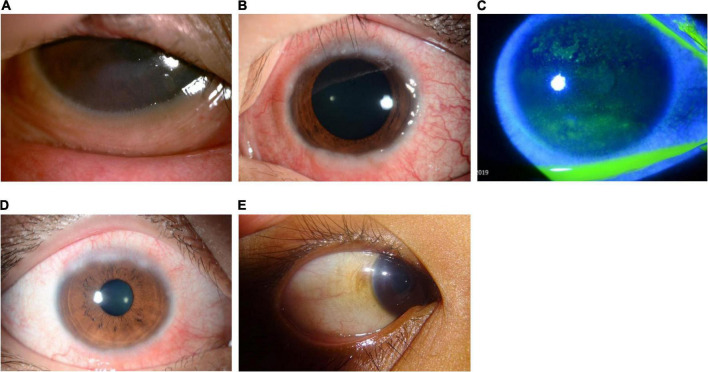
Clinical subtypes of VKC: *Limbal form*. The limbal form typically involves Horner–Trantas dots (see Figure 5), indicating lymphocytic and eosinophilic infiltration of the limbal conjunctiva. The mixed form is characterized by the presence of both tarsal and limbal subtypes in only one eye. (A) Image courtesy of Wei-Li Chen; (B–D) images courtesy of Jodhbir S. Mehta; (E) image courtesy of Florence Manurung.

Clinical signs of VKC include a papillary reaction of the upper tarsal conjunctiva and throughout the limbus. The disease can be classified into three clinical subtypes based on the location of the papillae: tarsal (palpebral; see Figure 2), limbal (bulbar; see Figure 3), or mixed form (3).

The tarsal form is characterized by large, cobblestone-like papillae on the upper tarsal conjunctiva. These can differ in shape and size, but are usually defined as >1.0 mm in diameter (1, 3). The limbal form typically involves Horner–Trantas dots, indicating lymphocytic and eosinophilic infiltration of the limbal conjunctiva (1, 3). The mixed form is characterized by the presence of both tarsal and limbal subtypes in only one eye (as signs are often heterogeneous between eyes) (1). According to the MOVIA Expert Working Group, the most common form of VKC seen in clinics across Asia is the tarsal form; however, up to one-third of patients are assumed to have the mixed form. The limbal form of VKC is considered less common in Asia (based on clinical experience).

#### Disease-Related Signs, Symptoms, and Complications

The most common ocular features of VKC are initially itching, redness, and tearing. Other common features include blurred vision, photophobia, burning, and a characteristic ropey, stringy mucus, and/or serous discharge (1, 16). Other typical signs and symptoms include moderate-to-intense conjunctival hyperemia, mild-to-moderate chemosis, foreign-body sensation, and pain, all of which can be very intense upon awakening, causing what is called “the morning misery” (1).

**FIGURE 4:**
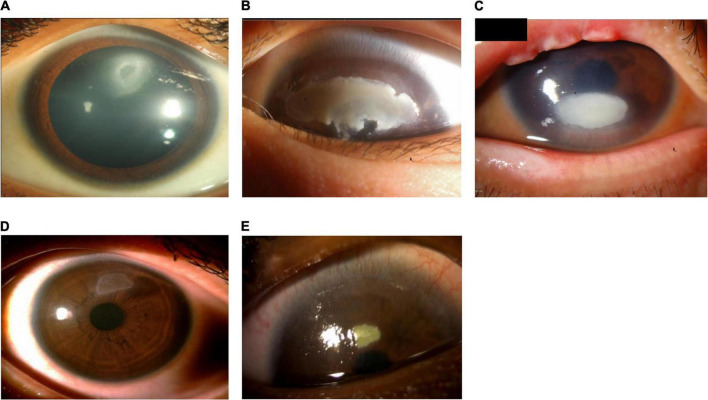
Patients with shield ulcer formation. Shield ulcers usually form on the upper third of the cornea. Plaques can also form when inflammatory debris accumulates at the base of a shield ulcer. (A) Image courtesy of Ramesh Kekunnaya; (B,C) images courtesy of Wei-Li Chen; (D) image courtesy of Leo Seo Wei; (E) image courtesy of Dominique Bremond-Gignac.

Severe VKC can result in sight-threatening complications. Ocular surface damage as a result of repetitive eyelid trauma and VKC-associated inflammatory activity can lead to corneal complications such as superficial punctate keratopathy (SPK), shield ulcers, corneal scarring, keratoconus, dry eyes, limbal stem-cell deficiency, and secondary infections (1, 6, 17, 18). Shield ulcers, which can be self-limiting or associated with bacterial keratitis, usually form on the upper third of the cornea, and can lead to loss of vision (see Figure 4) (3). Plaques can also form when inflammatory debris accumulates at the base of a shield ulcer (17), and can be particularly resistant to topical therapy or require surgical intervention (3). Limbal stem-cell deficiency can occur with longstanding inflammation (3). Keratoconus and irregular astigmatism can result from frequent eye rubbing in the pediatric population (10, 19, 20). In these patients, there is a fine balance between the benefits of corticosteroids and the risk for vision loss as a result of overtreatment. Patients with severe VKC may also develop lid complications and acquire ptosis (often with atopic dermatitis). The variable severity of these complications in each patient presents a challenge to ophthalmologists to not only manage acute episodes, but also to prevent reappearances.

Despite the limbal form of the disease being rare in Asia, a new clinical sign of VKC has been reported in patients from India and China—perilimbal hyperpigmentation. In these case reports, patients with VKC (ranging across all grades of disease, including remission) were observed to have fine, golden brown spotty pigmentations, mostly located in the perilimbal bulbar conjunctiva. With abundant melanocytes and mast cells around the limbus and complex immune mechanisms involved in VKC, it may be that any perilimbal conjunctival pigmentation is a by-product of these interactions and pathways. Whether environmental or racial factors have a role in the presence and/or distribution of the pigmentation remain to be solved. Immunopathological studies of conjunctival specimens from patients with VKC may provide additional information. More research is required to understand whether this sign is a consistent clinical finding, and whether this may be a useful diagnostic sign in patients with early or mild disease when signs and symptoms are subtle (21).

VKC usually resolves after puberty or adolescence. This means VKC can evolve and even spare the cornea, with only occasional presentation of symptoms during seasonal periods. However, without adequate treatment, the seasonal form can evolve into a chronic perennial inflammation after a mean of 3 years from disease onset (4). By contrast, children diagnosed with AKC will suffer from manifestations throughout their life, and may develop more severe complications, as the condition is progressive (22).

#### Burden of VKC

VKC is associated with a substantial impact on quality of life and daily living. Symptoms, which can be exacerbated by exposure to allergens or irritant stimuli, can lead to sleep deprivation and the inability to be outdoors (23). The latter has significant negative consequences for the daily life and social interactions of children, including playing sports, meeting friends, and attendance at school.

VKC also negatively impacts quality of life in adult patients (24). An increased disease duration and considerable inflammatory state (atopic sensitivity) associated with adult VKC can significantly impact the life and daily activities of individuals, with an additional economic burden (24). In patients with severe VKC, psychological support may be required.

Inadequate counseling and unrealistic expectations, often resulting in the overuse, misuse, and self-use of corticosteroids, can be associated with complications. Overmedication with corticosteroids can cause vision loss, as can undermedication (although uncommon) and persistent inflammation resulting in corneal scarring and stem-cell damage (25). The long-term use of corticosteroids by patients is often unsupervised and can be associated with raised intraocular pressure (IOP) and glaucoma, cataracts and opportunistic infections (10, 18–20, 26). Patients who are overtreated with corticosteroids may live with vision impairment for years, with an increased financial burden to treat the aforementioned complications (20).

### Rationale and Objectives

The aim of these expert-led recommendations is to provide general ophthalmologists in Asia with guidance for the diagnosis and management of VKC in order to inform clinical decisions and improve patient outcomes. The recommendations aim to incorporate the best available evidence into clinical practice to close the gap between the current standard of care and what the literature supports. The management recommendations are intended to progress clinical practice across Asia from a “conventional” approach to an evidence-based paradigm to provide patients with the greatest disease control as early as possible.

## Methods

The recommendations and information stated herein are based on the best available evidence as interpreted by an expert panel of 12 ophthalmologists from across Asia (Hong Kong SAR, India, Indonesia, Malaysia, Philippines, Singapore, South Korea, Taiwan, Thailand, and Vietnam) and one expert from France, all of whom have specialism and interest in the ocular surface, VKC, and pediatric ophthalmology. Gaps were identified and recommendations suggested by the expert group based on clinical expertise and available literature at the time of writing. The consensus program and development of this article followed an iterative process, led by two co-chairs of the MOVIA Expert Working Group (Prof. Jodhbir S. Mehta and Prof. Dominique Bremond-Gignac), with regular review and input from all members. Consensus was confirmed in a MOVIA Expert Working Group meeting held in October 2020 with every member of the group formally agreeing on the recommendations and paradigm presented. Voting included a five-way Likert scale to ascertain the certainty and strength for each—comprising the following options: “Weak” (associated with a score of –1), “Neutral” [0], “Moderate” [1], “Strong” [2], and “Very strong” [3]. The values of the votes were averaged, and the strength of the recommendation determined—with scores ≥2.5 were categorized as “Very strong” [+++]; ≥1.6 to <2.5 as “Strong” [++]; and ≥0.8 to <1.6 as “Moderate” [+]. Any recommendations with score <0.8 were not included.

## Unmet Needs Identified

The MOVIA Expert Working Group identified key unmet needs, including:

• Diagnosis and treatment of patients with VKC—this is a challenge for many ophthalmologists, since no precise diagnostic criteria have been established, the pathogenesis of the disease is unclear, and anti-allergic treatments are often ineffective in patients with moderate or severe disease (27).
• Choice of treatment and management strategies—currently, those used for patients with the same severity of disease varies greatly from country to country and physician to physician across Asia. This is because of a lack of well-defined, standardized guidelines and grading systems (25).
• Safety and iatrogenic complications (28).
• Optimal dosing regimens of pharmacological treatments (28), including supervision and compliance of readily available options.

### Providing Clarity in the Diagnosis of VKC

VKC forms part of a spectrum of ocular allergic disorders—termed allergic conjunctivitis—that affect the eyelid and conjunctiva (14). Other clinical forms that belong to this collection of diseases include contact blepharoconjunctivitis (CBC), seasonal allergic conjunctivitis (SAC), perennial allergic conjunctivitis (PAC), AKC, and giant papillary conjunctivitis (GPC) (14). Corneal involvement is typically restricted to the two most severe forms of ocular allergy, VKC and AKC, which require particular care in their diagnosis and management, with careful documentation of the patient’s clinical history and the use of slit-lamp examination for key clinical signs (28–30). The diagnosis has to be made as early as possible in order to specify the individual’s prognosis: regression in VKC (usually disappearing at adolescence) and progression in AKC (into adulthood) (31).

**FIGURE 5:**
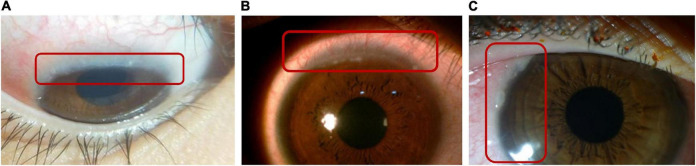
Horner–Trantas dots. Peri-limbal Horner–Trantas dots are focal white dots consisting of degenerated epithelial cells and eosinophils and are indicative of VKC. (A) Image courtesy of Florence Manurung; (B) image courtesy of Leo Seo Wei; (C) image courtesy of Dominique Bremond-Gignac.

**FIGURE 6:**
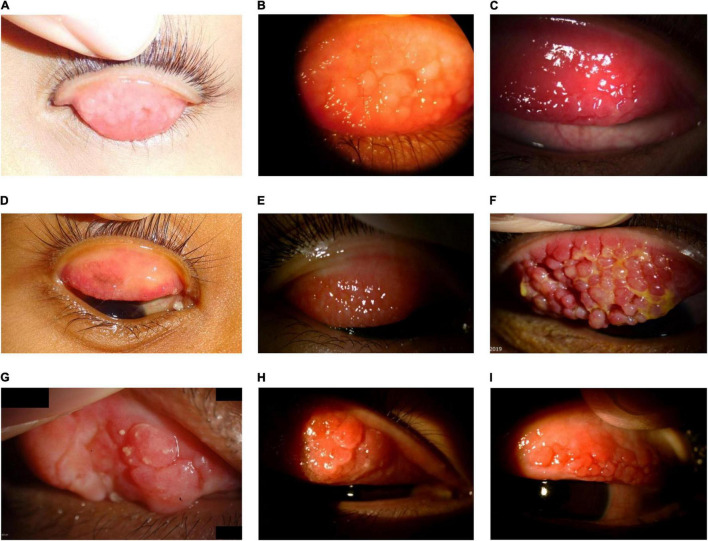
Large cobblestone papillae. Upper tarsal giant papillae are typical of VKC. These have characteristically flattened tops which sometimes demonstrate stain with fluorescein. Giant papillae can sometimes be seen near the limbus and, while relatively uncommon, symblepharon formation and conjunctival fibrosis can occur. (A–D) Images courtesy of Florence Manurung; (E) image courtesy of Douglas K. Lam; (F) image courtesy of Jodhbir S. Mehta; (G) image courtesy of Wei-Li Chen; (H-I) images courtesy of Leo Seo Wei.

While the differential diagnosis of diseases belonging to the allergic conjunctivitis family can be challenging, VKC is relatively easy to diagnose by clinical examination. Horner–Trantas dots (see Figure 5) and large cobblestone papillae (see Figure 6) are indicative of the disease (1, 3). On the other hand, AKC in children can be misdiagnosed as the presentation appears similar. However, atopic dermatitis or eczema often accompany this ocular allergy, with signs of thickened dry skin, and can be used to differentiate AKC from VKC. Particular attention can be given at first examination to the extent of atopic dermatitis across the skin, elbow, neck or canthus (31).

VKC can be differentiated from other ocular allergic conditions or drug-induced conjunctivitis (which can occur following instillation of eye drops, e.g., topical antiglaucoma agents, mydriatics, or alpha-adrenergic agonists commonly used as decongestants in over-the-counter anti-allergy eye drops) through a comprehensive clinical history and ophthalmic examination (28). No single clinical feature viewed in isolation can accurately differentiate AKC and VKC—a multifactorial assessment of key clinical signs presented, taking into consideration the presence of AKC-specific clinical features and the absence of VKC-related clinical features, in combination with a history of eczema and conjunctivitis/keratitis, may promote accurate diagnosis in children (31).

Table 2 includes clinical features that can assist the differential diagnosis of VKC vs. other common forms of ocular allergy. The diagnosis is generally based on the signs and symptoms of the disease, but in difficult instances can be aided by conjunctival scraping to demonstrate the presence of infiltrating eosinophils (4). Recently published guidelines in Japan include a diagnostic flowchart for allergic conjunctival diseases (in keeping with Table 2) which highlights key diagnostic differentiators for VKC, AKC SAC, PAC, and GPC. These include the absence or presence of conjunctival proliferation, seasonality of the condition, use of contact lenses, and absence or presence of atopic dermatitis, alongside the common symptom of ocular itching and hyperemia (32).

**TABLE 2:** Clinical features of major ocular allergy syndromes, including the underlying hypersensitivity mechanism and ophthalmological presentation (68).

	VKC	AKC	CBC	GPC	PAC	SAC
**Clinical presentation**	Persistent ± intermittent exacerbations	Chronic	Chronic ± intermittent exacerbations	Persistent	Persistent	Intermittent
**Allergic mechanism(s)**	IgE- or non-IgE-mediated	IgE- or non-IgE-mediated	Non-IgE-mediated	Non-allergic	IgE-mediated	IgE-mediated
**Background**	**Childhood** ± atopic	Adult atopic	Non-atopic	Atopic or non-atopic	Atopic	Atopic
**Eyelids**	Edema, pseudoptosis	Eczema + meibomitis, blepharitis, Dennie–Morgan folds	Erythema, eczema	–	± Edema	Edema
**Conjunctiva**	**Giant papillae, hyperemia**	Papillae ± fibrosis	± Hyperemia, follicles	Giant papillae	Follicles and/or papillae	Follicles and/or papillae
**Limbus**	± Thickened + **Horner–Trantas dots**	± Thickened ± Horner–Trantas dots	–	Hyperemia	–	–
**Cornea**	SPK ± shield ulcer ± vernal plaque ± keratoconus	SPK, shield ulcer, plaque, opacities, scars, neovascularization ± keratoconus	–	Rare	–	–
						

*Clinical features in bold are key differentiators from other ocular allergic conditions. Adapted from Leonardi et al. 69. AKC, atopic keratoconjunctivitis; CBC, contact blepharoconjunctivitis; GPC, giant papillary conjunctivitis; PAC, perennial allergic conjunctivitis; SAC, seasonal allergic conjunctivitis; SPK, superficial punctate keratopathy; VKC, vernal keratoconjunctivitis.*

The MOVIA Expert Working Group highly recommend the use of slit-lamp examination and the inversion and examination of the eyelids in the initial diagnostic approach for VKC, as many of the key clinical features appear on the eyelid or palpebral conjunctiva. Fluorescein staining may also help to identify sight-threatening corneal involvement in patients with moderate or severe disease.

Outside of ocular allergy and drug-induced conjunctivitis, other key differential diagnoses include:

• Pediatric blepharokeratoconjunctivitis—commonly misdiagnosed as ocular allergy in children of the same age range who present with blepharitis (33).
  ° This can be differentiated from AKC and VKC through assessment of symptomology and corneal changes (31). It is important to consider that in patients with blepharokeratoconjunctivitis, crusting of the lids may ensue in the morning, but discharge is not a major feature. In addition, there is frequently a history of recurrent chalazia. However, the condition may occasionally be asymptomatic until the presentation of photophobia, reduced vision and corneal opacities. Notable corneal changes in blepharokeratoconjunctivitis can include punctate epithelial erosions, peripheral (or marginal) keratitis, central or paracentral opacities with and without scarring, and secondary corneal vascularization (34).
  ° The presence of subconjunctival (cholesterol) oil crystals may also be a specific sign for blepharokeratoconjunctivitis (35).
• Infectious ulcers—the prevalence of which is anecdotally increasing among children in Asia due to the occurrence of orthokeratology lens-related corneal infections and trauma (36).
  ° It is important to differentiate ulcers from VKC, as treatment with corticosteroids can worsen the condition.
• Scleritis (episcleritis).
  ° Look for localized or widespread edema and purplish erythema, which indicate a serious cause, such as scleritis.
• Chlamydial infection (or trachoma)—distinguished by the presence of tarsal scarring (line of Arlt) and scarring of limbic follicles (Herbert’s pits).
• Corneal foreign body—where removal can leave acute epithelial defects.

Slit-lamp examination and eyelid eversion should allow the identification of clinical features related to VKC. The following areas should be prioritized to understand the nature of the ocular allergy:

• Conjunctiva—papillae (small conjunctival elevations with central vessels), follicles, chemosis (swelling), and membranes. Pull on the lower eyelid and evert the upper lid to examine the palpebral conjunctiva.
• Cornea—look for SPK, ulceration, opacities, and Horner–Trantas dots. Keratoconus and astigmatism, as well as vernal plaques, may present in patients with moderate-to-severe VKC or AKC.
• Eyelids—look for discharge, swelling, inflammation, loss of lashes (may present in VKC and AKC), lice infestation, or blepharitis.
• A dull blue discoloration below the eye (sometimes called the “allergic shiner”) resulting from venous congestion may be present in some people with allergies. This is often related to allergic rhinitis and should not be confused with Dennie–Morgan folds in the lower eyelid, which is a common sign of AKC.

Supplementary ophthalmic examinations, although non-essential in the context of ocular allergy assessment, include:

• Visual acuity—compare with previous levels of visual acuity, if possible.
• IOP—it remains important to monitor IOP in a condition where steroids may be used and associated with IOP-increasing adverse events.

If available, tear film stability and breakup patterns can be assessed using fluorescein application, and the cornea can be examined for abnormalities, such as small branching corneal dendrites (characteristic of herpes simplex keratitis), single or multiple dendrite-like epithelial or subepithelial lesions (pseudodendrites), and corneal ulcers. VKC should also be assessed with respect to dry eye disease, as the two conditions often coexist (1).

Adjunctive tests should be considered in the context of a multidisciplinary team approach, if needed and locally available. These include skin-prick tests, blood tests, examination by allergists (for asthma, dermatitis, rhinitis, eczema), and conjunctival allergen provocation tests. Two VKC populations can be defined according to the diagnostic criteria:

1. Those with positive test results, who generally also present with some other allergic manifestation, such as asthma, rhinitis, or eczema;
2. Those with negative test results, and a negative personal and familial history of atopy.

Importantly, while skin-prick tests are useful for allergen detection (and results may be positive), VKC is not always closely related to allergen exposure, and climate is an equally critical factor (1).

Conjunctival scrapings or tear cytology may be useful to indicate increased leukocytes in the conjunctiva, particularly eosinophil levels (1). However, biochemical markers are not a requisite for diagnosis. In many cases, patients should be referred to a corneal specialist or specialist pediatrician to receive treatment without the need for full diagnosis (1, 30).

Research has suggested that dendritic cells play an essential role in VKC, with higher numbers reported in patients with VKC compared with normal eyes. The dynamic changes of dendritic cells at the conjunctiva and cornea, such as density, morphology, and distribution, may be observed with *in vivo* confocal microscopy (37). This could help the assessment, diagnosis, and monitoring of the disease. However, the application in everyday practice across Asia may be limited to specialists and/or research purposes, due to access and availability.

### Current Practice in Clinical Grading and Assessment of Severity

Symptomatic or clinical grading criteria can provide general ophthalmologists (and pediatricians) with clearly defined parameters that can guide referral of the patient to a corneal specialist or allergist for diagnostic confirmation (7).

**FIGURE 7:**
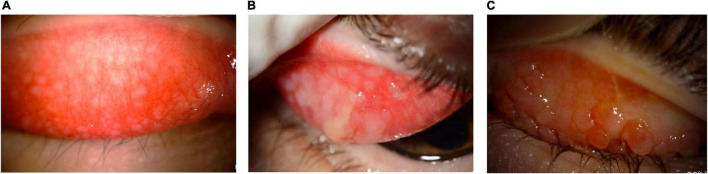
Characteristic papillary hyperplasia in VKC. The conjunctiva often shows hyperplasia, with infiltration of lymphocytes and eosinophils. **(A)** Image courtesy of Wei-Li Chen; **(B)** image courtesy of Jodhbir S. Mehta; **(C)** image courtesy of Dominique Bremond-Gignac.

The finding of papillary hyperplasia (see Figure 7) is mandatory for the diagnosis of VKC, and there is agreement on classifying the disease based on the part of the conjunctiva involved (tarsal, limbal, or mixed form). Papillae are variable in size, ranging from 0.1 to 5.0 mm in diameter (7). However, there is no consensus on the threshold that distinguishes giant from small papillae, with proposed cut-offs ranging from 1.0 to 3.0 mm in diameter (7).

With regard to the severity of the disease, some models of combined evaluation of symptoms have been proposed. Across Asia, the most widely used model is the Bonini scale (27), on which the most frequent grades of VKC seen in ophthalmology clinics is understood to be moderate and severe.

**FIGURE 8:**
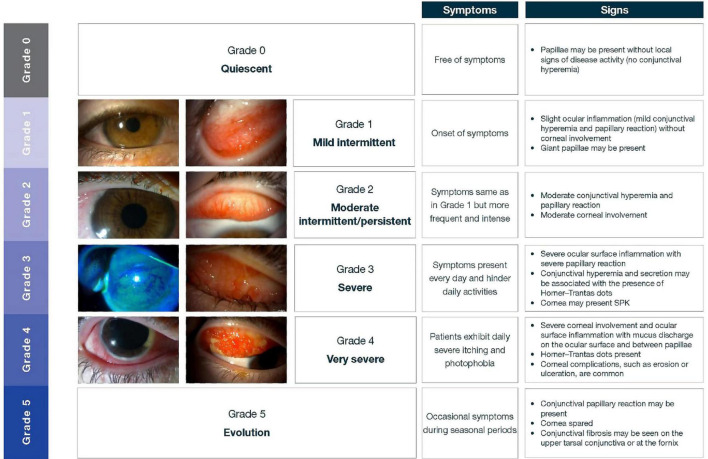
Levels of severity of VKC, based on the Bonini grading scale. Adapted from Bonini et al. (27). Images courtesy of Dominique Bremond-Gignac. SPK, superficial punctate keratopathy.

This Bonini grading is based on the clinical signs and symptoms of ocular surface inflammation to help classify the severity of disease (7, 27). Figure 8 outlines this grading system.

A variety of other grading schemes are available but are not as widely used in Asia. Pucci et al. developed a grading score of subjective ocular symptoms (itching, photophobia, tearing, foreign body, and burning sensation) ranging from 0 (no symptoms) to 15 (severe clinical picture) (38). Several other grading schemes may be available to estimate corneal involvement in VKC, such as the new VKC-Collaborative Longitudinal Evaluation of Keratoconus study (VKC-CLEK) system, which aims to better evaluate limbal and tarsal epithelial damage in patients with VKC (39). Although the Bonini scale is in common use across Asia, many ophthalmologists simply use personal clinical notetaking to assess the progression of the condition and treatment effects.

The severity of VKC is often used as an indicator for treatment escalation, where no improvement in symptoms or signs within 2–4 weeks results in a step up in the management approach.

### Coming to Consensus for the Management of VKC

#### Non-pharmacological Management

Where possible, first-line management of VKC should involve the identification of relevant allergens and avoidance of non-specific environmental factors that aggravate the disease (1), particularly during periods of exacerbation, such as sunlight, wind, and salty water. Frequent hand, face, and eye washing, alongside lid hygiene, is also strongly recommended (28). Furthermore, artificial tears (ocular lubricants) can aid in stabilizing the tear film to provide a better mucosal barrier against allergens, acting as an eyewash and diluting the concentration of mediators in the tear film in contact with the ocular surface (28). Products with herbal extracts, such as chamomile-containing eye drops, should be avoided as they may cross-react with allergens (e.g., *Artemisia vulgaris*) (28). Cold compresses are also recommended for use as a decongestant (1, 28).

Patients and caregivers should be given educational support regarding the anticipated duration and prognosis of the disease (see Table 3), and the possible complications from suboptimal control. In addition, it must be made clear that unsupervised or overuse (self-medication) of certain treatments for VKC, such as corticosteroids, must be avoided as this can lead to impaired vision or blindness (25, 26). Psychological support may be necessary for patients with severe VKC.

**TABLE 3:** Summary of patient education and preventive measures in the management of VKC.

**Patients/caregivers should be advised that:**
• VKC is a chronic, recurrent condition that usually improves with age.
• Rubbing itchy eyes can make the condition worse.
• Sunlight, wind, salty water, dust, and heat can exacerbate VKC, so the use of sunglasses, hats, visors, and swimming goggles should be considered.
• Common allergens can exacerbate VKC. Frequently washing hands, face, and hair can reduce exposure to these allergens.
• Cold compresses and preservative-free artificial tears can provide symptomatic relief.
• Vacations in locations with unsuitable climates should be avoided.
• An air-filtration system in the home may provide relief.

Where possible, a collaborative approach between the family doctor and medical specialists should be considered (1). VKC often requires a multidisciplinary approach—immunologists, pediatricians, and allergists should all be consulted as and when appropriate (1). Despite varying considerably between and within Asian countries, access to care remains important for patients with allergies to receive an assessment promptly to manage any seasonal flares, rather than refer to self-medication.

#### Pharmacological Management

Controlling the signs and symptoms of VKC can be a challenge for even experienced ophthalmologists. Given the chronicity and severity of the disease, non-pharmacological treatment options, such as avoiding specific and non-specific triggers and lifestyle planning, should be accompanied by pharmacological treatments (1).

This article includes a comprehensive overview of the currently approved treatments for VKC; however, it may not include all therapies that are currently available in Asia, under evaluation, or in the early stages of research. Available topical drugs include antihistamines, mast cell stabilizers, dual-acting agents (combination of antihistamines and mast cell stabilizers), alpha-adrenergic agonists (vasoconstrictors), non-steroidal anti-inflammatory drugs (NSAIDs; prostaglandin inhibitors), immunomodulators [e.g., cyclosporine A (CsA)], and corticosteroids. Table 4 summarizes some of the available and commonly used treatments (28). Additional information on available treatments and dosing by region are provided in Supplementary Tables 1–6.

**TABLE 4:** Overview of pharmacological options currently available across Asia for the management of VKC (28)*.

Class	Drug	Standard dosing	Indication	Considerations
**Topical antihistamines** (second generation)	Antazoline Emedastine Levocabastine	QID	Relief of itching Relief of signs and symptoms	• Short duration of action • Frequently not enough alone to treat the entire disease
**Mast cell stabilizers**	DSCG Lodoxamide NAAGA Pemirolast potassium	BID to QID	Relief of signs and symptoms	• Long-term use • Slow onset of action • Prophylactic dosing • Frequently not enough alone to treat the entire disease
**Dual-acting agents** (antihistamine + mast cell stabilizers)	Alcaftadine Azelastine Bepotastine Epinastine Ketotifen Olopatadine	QD QD to BID BID BID BID to QID BID	Relief of itching Relief of signs and symptoms	• Side effects • Bitter taste (azelastine) • Frequently not enough alone to treat the entire disease
**Immunomodulators** (calcineurin inhibitors)	Cyclosporine A Tacrolimus	QD to QID QD	Treatment of severe VKC and AKC not responding to anti-allergic drugs	• Pharmacy-compounded preparations vary from center to center • Quality control and availability of pharmacy-compounded preparations are poor • Tacrolimus is largely used off-label in ocular allergy (tacrolimus is approved for VKC only in Japan)
**Corticosteroids**	Betamethasone Desonide Dexamethasone Fluorometholone Hydrocortisone Loteprednol Prednisolone Rimexolone	As required (up to 7 days)	Treatment of allergic inflammation Use in moderate-to-severe forms	• Risk for long-term adverse events • No mast cell stabilization • Potential for inappropriate patient use • Requires close monitoring
**Vasoconstrictors** (vasoconstrictor–antihistamine combinations)	Naphazoline/ pheniramine	BID to QID	Episodic itching and redness	• Rapid onset but short duration of action • Only addresses hyperemia • Associated with a range of side effects • Potential for inappropriate patient use

*The table was correct as of January 2021.*

*Availability and access to treatments may vary across clinics, hospitals, regions, and countries. Each treatment option should be considered in accordance with the level of evidence available at the time.

*AKC, atopic keratoconjunctivitis; BID, twice daily; DSCG, disodium cromoglycate; NAAGA, N-acetyl-aspartyl glutamic acid; QD, once daily; QID, four times daily; VKC, vernal keratoconjunctivitis.*

In patients with a history of VKC, pharmacological treatment should be planned early and started at the beginning of spring or continued all year, depending on allergen exposure and duration of symptoms (1). While treatment approaches may differ in regions or countries in Asia with seasonal variation, specific treatment recommendations should depend on the disease severity and cause. The sequence and combination of therapies should be determined on the patient’s needs and preferences, and the treating ophthalmologist’s medical judgment. Any treatment decisions should be made on an individual patient basis after evaluating the benefits and risks of available therapies.

Figure 1 summarizes the MOVIA Expert Working Group’s recommended stepwise management approach according to disease severity. For the use of any eye drops on the ocular surface, it is recommended to use preservative-free compounds, where possible, to minimize ocular surface toxicity. The pharmacological strategies presented here align with that presented in the recent Japanese guidelines for allergic conjunctival diseases (32).

##### Conventional Topical Treatments

*a. Antihistamines*

Antihistamines act *via* histamine receptor antagonism to block the inflammatory effects of endogenous histamine and relieve any associated signs and symptoms. Most antihistamines used in the treatment of ocular allergy are H1 receptor antagonists, although some agents have affinity for other subtypes. H2 antagonists have been shown to modulate both cell growth and migration. Animal model studies have shown that antihistamines may even reduce infiltration of eosinophils and thus reduce the clinical aspects of the late-phase reaction (1).

Overall, topical antihistamines (instilled four times daily) appear to be safe and well tolerated in patients with VKC. Alongside mast cell stabilizers and dual-acting agents, they are often the first-choice treatment and are effective in reducing the signs and symptoms of VKC (28). Antihistamine drugs with ocular activity are a good therapeutic option for patients with allergic conjunctivitis, including VKC, since they can inhibit pro-inflammatory cytokine secretion from conjunctival epithelial cells (40).

First-generation antihistamines are well tolerated and associated with a favorable long-term safety record, but are associated with instillation pain, short duration of action, and limited potency (41). They remain available in over-the-counter products, particularly in combination with vasoconstrictors. While newer antihistamines are also H1 antagonists, they have a longer duration of action (4–6 hours) and are better tolerated than their predecessors (1).

*b. Mast cell stabilizers*

Alongside antihistamines and dual-acting agents, mast cell stabilizers (instilled two to four times daily) form the first line of treatment and are effective in reducing the signs and symptoms of disease. Mast cell stabilizers have been shown to act on multiple cells involved in allergic inflammation, including eosinophils, neutrophils, macrophages, mast cells, and monocytes. For example, lodoxamide is known to have an effect on eosinophil activation (shown in studies by measuring tear eosinophil cationic protein before and after therapy), while N-acetyl-aspartyl glutamic acid (NAAGA) is known to inhibit leukotriene synthesis and release of histamine by mast cells (1).

Overall, they are generally well tolerated (especially preservative-free options); however, there are tolerability concerns with some agents, including burning and stinging sensations upon instillation, blurred vision, and an undesirable aftertaste. Data on long-term efficacy and safety are lacking (28).

*c. Dual-acting agents*

The combination of mast cell stabilizing properties and histamine receptor antagonism (instilled twice daily) has shown clear benefits in treating forms of ocular allergy. A key benefit of these agents is the provision of rapid symptomatic relief by alleviating itching and redness (1). Olopatadine and ketotifen, for example, have shown to be effective in relieving itching, tearing, conjunctival hyperemia, mucus discharge, and photophobia (42).

Dual-acting agents are well tolerated and are not associated with significant ocular drying effects (1), and should be preferred over monotherapy with antihistamines or mast cell stabilizers alone.

### Recommendations

• All of the topical anti-allergic drugs mentioned here are effective for reducing signs and symptoms of mild or moderate VKC [++].
• Dual-acting agents should be considered ahead of monotherapy with antihistamines or mast cell stabilizers [+++].

#### Topical Immunomodulators

Topical calcineurin inhibitors are frequently prescribed for patients with VKC (28). They are recommended in patients with acute-phase or persistent, moderate or severe VKC that is not responding to anti-allergic drugs (28).

*a. Cyclosporine A*

Topical CsA is effective in controlling ocular surface inflammation in VKC and is thought to work by inhibiting Th2 proliferation and IL-2 production, and by reducing levels of immune cells and mediators acting on the ocular surface and conjunctiva (25).

Different concentrations of CsA, ranging from 0.05 to 2.0%, are currently available in different countries with different clinical indications (43, 44). The 0.1% cationic emulsion (CE) formulation increases the bioavailability of CsA in the tear film compared with available oil-based (often used when CsA is pharmacy-compounded) or anionic emulsion CsA formulations (45). Clinical studies on the efficacy of topical CsA 0.1% CE for treating VKC have consistently shown a beneficial effect of the drug, with safety and efficacy demonstrated for up to 7 years and comparable with those seen in patients with dry eye disease (25, 46–48).

There is no consensus on the minimum effective concentration of CsA to treat VKC (1), as this can depend on the emulsion vehicle. Currently, CsA 0.1% CE is approved based on significant improvements in signs, symptoms, and quality of life in patients with severe VKC, and is commercially available across Asia. It is recommended that CsA 0.1% CE should be initiated twice daily for patients with mild-to-moderate disease and four times daily for those with moderate-to-severe disease for up to 6 months. Other commercially available CsA formulations exist but are not approved for use in VKC (as of December 2020). In addition, CsA may be compounded in hospital-based pharmacies to provide variable concentrations and formulations; however, its quality may be compromised, and availability is limited to specialist centers.

The most commonly associated adverse event with CsA is a stinging or burning sensation on instillation, and no new safety issues were identified in long-term follow-ups (25, 46–48). Instillation pain can be minimized by following a standard technique for ophthalmic drop instillation, which includes using artificial tears prior to instillation (avoiding any potential washout effect of the subsequently administered CsA, by waiting at least 10 minutes between administrations), followed by one drop into the lower conjunctival sac of each eye in the morning, at noon, in the afternoon, and in the evening, approximately 4 hours apart (48). Patients should also be counseled to expect stinging and burning when the drops are applied, but that the sensation should taper off with regular use as prescribed.

After long cycles of treatment, CsA can allow control of symptoms without corticosteroids. There is a trend in the Asia-Pacific region, as shown by these expert-led recommendations, in using CsA as a first-line treatment for patients who present with moderate-to-severe or persistent VKC. In patients presenting with conjunctival involvement only, CsA alone is recommended for treatment, while corticosteroids should only be prescribed (as short pulses) if there is an inadequate response to CsA. Otherwise, CsA can be used in combination with corticosteroids in patients with corneal complications (or limbitis) or acute exacerbations, in the understanding that CsA may take over a week to act due to its immunosuppressive mechanism of action.

*b. Tacrolimus*

Tacrolimus is a strong, non-steroidal immunosuppressant that binds to FK506-binding proteins in T lymphocytes and inhibits calcineurin activity (49). Tacrolimus has been investigated in a small randomized controlled trial in VKC, where it demonstrated improvements in symptoms of itching, foreign-body sensation, and photophobia, as well as in signs of limbal inflammatory activity and keratitis, with maintenance of disease control (50, 51). Tacrolimus may be administered as eye drops or an ointment; however, the eye drop formulation is not approved for use in many countries in Asia. The use of tacrolimus (0.03%) ointment in ophthalmic and dermatologic form is currently off-label in Asia for patients with VKC. However, there are findings in published studies of small population sizes demonstrating effectiveness and tolerability (50–55).

Similar to CsA, tacrolimus may be associated with instillation pain (49). As a strong immunosuppressant, topical tacrolimus may be associated with an increased risk for corneal infections with prolonged use, and close monitoring of patients on long-term therapy is necessary (55). Topical tacrolimus (0.02–0.1%) should be reserved for patients with severe VKC involving allergy of the eyelid (in countries where it is currently available) or whose disease is refractory to CsA (28).

### Recommendations

• Topical CsA 0.1% CE should be considered for patients with moderate-to-severe or persistent VKC [+++].
• Patients should be instructed on how to apply CsA eye drops to minimize stinging or burning on instillation, such as using artificial tears prior to instillation [+++].
• In regions where available, tacrolimus should be reserved for patients with severe VKC that is refractory to CsA. It can be considered as a treatment for moderate-to-severe VKC in patients with allergy of the eyelid, but please note this may be off-label [++].

#### Corticosteroids

Topical corticosteroid eye drops should be used as short, pulsed therapy to provide symptomatic relief in patients with more persistent VKC or acute exacerbations, or when the cornea is involved (e.g., patients with shield ulcers or hyperplasia in limbal VKC), and under an ophthalmologist’s monitoring (28). Topical corticosteroids can be administered at a low or high dose, depending on the severity of VKC, and can be given up to four times daily with or without CsA. Corticosteroids act by suppressing the late-phase reaction in both experimental and clinical settings. They, in part, limit the inflammatory cascade by inhibiting phospholipase A2, and consequently act to prevent migration of leukocytes, release of hydrolytic enzymes, growth of fibroblasts, and lead to changes in vascular permeability (56).

Moderate-to-severe VKC may require repeated topical corticosteroid treatment to downregulate conjunctival inflammation (1). Persistent and severe symptoms, thick mucus discharge with moderate-to-severe corneal involvement, numerous and inflamed limbal infiltrates, and/or giant papillae may indicate a need for corticosteroids in addition to CsA use (1). However, use of corticosteroids as first-line therapy is not recommended in those with only conjunctival involvement (1).

Corticosteroids with low intraocular absorption (“soft steroids”), such as hydrocortisone, fluorometholone and loteprednol, may be preferred (1). Dosages are chosen based on the inflammatory state of the eye, with therapy prescribed in pulses of 3–5 days (1). Loteprednol is usually indicated for 7–8 days in the treatment of the acute phase (1). Prednisolone, dexamethasone, and betamethasone are sometimes considered only as second-line options, or as first-line treatment in more severe cases, due to their potential effect on intraocular pressure (1). Steroid–antibiotic combination eye drops are not recommended, since VKC is an allergic inflammation and not an infection (1).

Corticosteroids are not recommended for long-term use because of the increased risk for ocular adverse events, including increased IOP, glaucoma, cataracts, and susceptibility to infection (1). These adverse events depend, in part, on the structure of the steroid, the dose, and duration of treatment (57). Treatment with corticosteroids requires careful monitoring for the development of IOP, which should be promptly brought under control to prevent deterioration or loss of vision (58).

Supratarsal corticosteroid injections have demonstrated some value in treating adults with VKC but may not be an appropriate approach for children or for application by general ophthalmologists (58, 59). For more specialist ophthalmologists, this may be an option in children with refractory, severe, or challenging VKC.

### Recommendations

• In patients with only conjunctival involvement, topical corticosteroids should be reserved for use after loss of control or persistence of symptoms with immunomodulators (such as CsA) [++].
• Topical corticosteroids are effective for the management of acute exacerbations, or when the cornea is involved, and preferably only introduced in patients with more severe disease. In these individuals, corticosteroids should be used in combination with CsA to account for the fact that CsA may require ≥1 week to act [+++].
• Because of an increased risk for adverse events and/or vision loss with chronic use, topical corticosteroid eye drops should be used in short pulses (alone or in combination with CsA) under the supervision of an ophthalmologist and tapered rapidly [+++].

#### Vasoconstrictors

Topical vasoconstrictors can be effective at alleviating hyperemia but offer little to no relief from itchiness and have a short duration of action. Vasoconstrictors may cause several side effects including rebound redness, chronic follicular conjunctivitis, and tachyphylaxis. Vasoconstrictors are rarely used in the pediatric population. It is recommended that these agents should be avoided. If used, they should be used with caution and only for a short period (no longer than 5–7 days) because of adverse events and tachyphylaxis (28).

Topical decongestants do not reduce the allergic response because they do not antagonize any of the mediators of allergic inflammation. Burning or stinging on instillation is a common adverse event, and prolonged use and/or discontinuation following longer-term use can lead to rebound hyperemia and conjunctivitis medicamentosa (60). These events are usually associated with topical combinations of vasoconstrictors and first-generation antihistamines, such as pheniramine and antazoline, which are available as over-the-counter products.

### Recommendations

• Vasoconstrictors are not recommended for the treatment of VKC [++].
• If used to address hyperemia, vasoconstrictors should be used with caution and only for a short period due to adverse events [+++].

#### Topical Non-steroidal Anti-inflammatory Drugs

Non-steroidal anti-inflammatory drugs (NSAIDs) used in the treatment of ocular allergy typically inhibit cyclooxygenase (COX)-1 and COX-2 enzymes (1). Some have demonstrated a slight effect in the treatment of ocular allergy, by targeting itching, intercellular adhesion molecule-1 expression, and tear tryptase levels (1, 28). However, use of NSAIDs is not encouraged in VKC because of their local side effects, such as burning or stinging after application, increased risk of inducing keratitis on the ocular surface, and because they do not target the specific inflammatory mechanism associated with VKC.

### Recommendations

• NSAIDs are not recommended as they do not target the specific inflammatory mechanisms associated with VKC [++].

#### Systemic Pharmacological Treatment

Systemic treatment with oral antihistamines or antileukotrienes can reduce the severity of flare-ups and generalized hyperreactivity (1). Newer second-generation antihistamines are preferred over older first-generation antihistamines, in order to avoid the sedative and anticholinergic effects that are associated with first-generation agents (61).

### Recommendations

• Second-generation systemic antihistamines are preferred over older first-generation antihistamines [++].

#### Allergen-Specific Immunotherapy

Allergen-specific immunotherapy is indicated only when a clearly defined systemic hypersensitivity to an identified allergen exists (28), and should be managed by an allergist or specialist ophthalmologist. The combination of clinical history with the results of a skin-prick test and specific serum IgE should be taken into consideration when the choice of immunotherapy is made (28). There are currently no robust studies of allergen-specific immunotherapy in VKC (28). A meta-analysis of several double-blind, randomized, placebo-controlled trials investigating sublingual immunotherapy for allergic conjunctivitis, reported a significant reduction in total ocular symptom scores and ocular signs (redness, itchiness, and tearing) vs. placebo in pollen-induced allergic conjunctivitis, but not in allergic conjunctivitis associated with house dust mites, in the pediatric population (62).

### Recommendations

• Allergen-specific immunotherapy is only recommended when clearly defined systemic hypersensitivity to an identified allergen exists [++].
• Patients requiring allergen-specific immunotherapy should be referred to an allergist or specialist ophthalmologist [+++].

#### Surgery

Case reports and ongoing clinical practice have indicated that, in rare instances, specialist surgical management may be beneficial in patients with VKC. Surgical approaches that focus on conjunctival cobblestone papillae, shield ulcers, corneal plaques, limbal insufficiency, and other ocular surface presentations that do not respond to medical treatment can be beneficial (63–65).

Surgical approaches can include excision of giant papillae, debridement of the corneal plaque to remove cytotoxic cells, and amniotic membrane transplantation (66). Surgical treatment, especially corneal plaque removal, may be appropriate for patients with ulcers of moderate-to-severe severity, because short-term re-epithelialization rates are higher and the number of complications is lower than that associated with medical therapy (66). Other reports suggest surgical treatment, such as giant papillae excision, may be appropriate in cases of corneal involvement and in the presence of coarse giant tarsal papillae resulting in ptosis (or mechanical pseudoptosis) (1, 67). Amniotic membrane transplantation following keratectomy has been described as a successful treatment for deep ulcers, in severe allergic patients with slight stromal thinning (68).

Patients with VKC who may benefit from surgical intervention should be referred to a corneal specialist first. Leonardi et al. have suggested that cryotherapy and/or giant papillae excision papillae should otherwise be avoided because they only treat the complications of VKC and not the underlying disease, and may induce unnecessary scarring (1).

## Conclusion

VKC is an underdiagnosed and underrecognized chronic form of ocular allergy that is an important public health problem in Asia, imposing a substantial burden on both patients and healthcare professionals managing their care. Chronic disease management remains a clinical unmet need; if inadequately treated, VKC can result in significant damage to the cornea and conjunctiva, with the potential for vision impairment. Adequate and continuous treatment through good patient education and regular, long-term follow-up are essential.

Treating VKC should entail a stepwise approach, identifying triggers, educating patients/caregivers on good ocular health, and addressing symptoms. In a new paradigm of management, the use of immunomodulators (e.g., topical CsA) should be considered early to tackle the inflammatory and chronic nature of VKC, with topical corticosteroids reserved as an add-on, short-pulse therapy for persistent disease, during acute exacerbations, or in patients with corneal involvement. Any use of corticosteroids requires tapering once symptoms have been controlled to avoid adverse events. For patients with an identified allergy, referral to an allergist is recommended for additional systemic treatment. In the rare patients who do not respond to medical treatment, surgery may be required.

## Disclosures

This article includes a comprehensive overview of the currently approved treatments for VKC; however, it may not be inclusive of all therapies for VKC that are currently available in Asia, under evaluation, or in the early stages of research. Availability of assessment tools, diagnostics, and treatments may differ between countries across Asia, as well as within regions and clinics. References to specific drugs, instruments, and other products are made for illustrative scientific purposes only and are not intended to constitute an endorsement of such. The expert-led recommendations are intended to generally meet the needs of most ophthalmologists and patients; they cannot possibly meet the needs of all. Any treatment decisions should be made on an individual patient basis after evaluating the benefits and risks of available therapies.

## Ethics Statement

All authors who have provided images for use in this article have obtained appropriate declarations of informed consent from the patient or legal guardian.

## Author Contributions

All authors were involved with the conceptualization, investigation, resources, validation, and writing (both original and draft presentation, review, and editing), and read and approved the submitted version of the manuscript. JSM and DBG provided supervision.

## Conflict of Interest

JSM was a consultant for, or has received research grants or travel grants from, Carl Zeiss, Cordlife, Leica, Millipore Sigma, Moria, Network Medical, Santen, Trefoil, and Ziemer. DBG was a consultant for Alcon/Novartis, Cooper, Hoya Surgical Optics, Santen, and Théa, and has received research grants or travel grants from Hoya Surgical Optics, Santen, and Théa. ACKC has received travel grants from Santen. TIK was a medical advisory board member for Hoya Surgical Optics and Santen. DKL has received travel grants from Johnson & Johnson Vision and Santen. SWL has received travel grants from Allergan, Carl Zeiss, and Santen. SWL was employed by Dr Leo Adult & Paediatric Eye Specialist Pte Ltd. NT was a medical advisory board member and educational speaker for Santen and has received travel grants from Santen and TRB Chemidica. The remaining authors declare that the research was conducted in the absence of any commercial or financial relationships that could be construed as a potential conflict of interest.

## Publisher’s Note

All claims expressed in this article are solely those of the authors and do not necessarily represent those of their affiliated organizations, or those of the publisher, the editors and the reviewers. Any product that may be evaluated in this article, or claim that may be made by its manufacturer, is not guaranteed or endorsed by the publisher.
