# Supplementary material for: Diagnosis, Management, and Treatment of Vernal Keratoconjunctivitis in Asia: Recommendations From the Management of Vernal Keratoconjunctivitis in Asia Expert Working Group
Source: Front Med (Lausanne). 2022 Aug 1;9:882240. doi: 10.3389/fmed.2022.882240 (PMC9376221; doi:10.3389/fmed.2022.882240)
Supplement: Supplementary file 1 [file Data_Sheet_1.docx]

Supplementary Material

**Supplementary Table 1.** Topical antihistamines available for VKC by country (provided by the MOVIA Expert Working Group).

| **Country/region** | **Drugs available** | **Dosing** |
| --- | --- | --- |
| Hong Kong | Emedastine 0.05% | QID |
| India | Emedastine 0.05% Levocabastine 0.05% | QID QID |
| Indonesia | N/A |  |
| Malaysia | Antazoline 0.5% (+ xylometazoline 0.05%) Levocabastine 0.05% | QID QID |
| Singapore | N/A |  |
| South Korea | N/A |  |
| Taiwan | Emedastine 0.05% | QID |
| Thailand | Antazoline 0.05% (+ tetrahydrozoline 0.04%) | BID to QID |
| Vietnam | N/A |  |

BID: twice daily; N/A: not available; QID: four times daily.
This table is correct as of January 2021.

**Supplementary Table 2.** Mast cell stabilizers available for VKC by country (provided by the MOVIA Expert Working Group).

| **Country/region** | **Drugs available** | **Dosing** |
| --- | --- | --- |
| Hong Kong | DSCG 2%  Lodoxamide 0.1% | BID to QID  BID to QID |
| India | DSCG 2% | BID to QID |
| Indonesia | DSCG 2%  Pemirolast potassium 0.1% | TID  TID |
| Malaysia | DSCG 2%  Lodoxamide 0.1% | BID to QID  BID to QID |
| Singapore | DSCG 2% | BID to QID |
| South Korea | DSCG 2%  NAAGA 49 mg/mL | QID  BID to six times daily |
| Taiwan | DSCG 2% | BID to QID |
| Thailand | DSCG 2%  Pemirolast potassium 0.1% | QID  BID |
| Vietnam | Lodoxamide 0.1% | TID |

BID: twice daily; DSCG: disodium cromoglycate; NAAGA: N-acetyl-aspartyl glutamic acid;
QID: four times daily; TID: three times daily.
This table is correct as of January 2021.

**Supplementary Table 3.** Dual-acting agents (antihistamines + mast cell stabilizers) available for VKC by country (provided by the MOVIA Expert Working Group).

| **Country/region** | **Drugs available** | **Dosing** |
| --- | --- | --- |
| Hong Kong | Alcaftadine 0.25%  Azelastine 0.05%  Epinastine 0.05%  Ketotifen 0.025%  Olopatadine 0.01–0.1% | QD  BID  BID  BID  BID |
| India | Alcaftadine 0.25%  Azelastine 0.05%  Ketotifen 0.025%  Olopatadine 0.1% | QD  BID  BID  BID |
| Indonesia | Olopatadine 0.1% | BID |
| Malaysia | Azelastine 0.05%  Epinastine 0.05%  Ketotifen 0.025%  Olopatadine 0.2% | QD to BID  BID  BID  QD to BID |
| Singapore | Alcaftadine 0.25%  Ketotifen 0.025%  Olopatadine 0.1% | QD  BID  BID |
| South Korea | Alcaftadine 0.25%  Azelastine 0.05%  Bepotastine 1.5%  Epinastine 0.05%  Ketotifen 0.069%  Olopatadine 0.1% | QD  QD to BID  BID  BID  BID to QID  QD |
| Taiwan | Epinastine 0.05%  Ketotifen 0.025%  Olopatadine 0.1% | BID  BID  BID |
| Thailand | Alcaftadine 0.25%  Epinastine 0.05%  Ketotifen 0.025%  Olopatadine 0.1% and 0.2% | QD  BID  BID  QD to BID |
| Vietnam | Epinastine 0.05%  Olopatadine 0.1% | BID  QD |

BID: twice daily; QD: once daily; QID: four times daily.
This table is correct as of January 2021.

**Supplementary Table 4.** Immunomodulators (calcineurin inhibitors) available for VKC by country (provided by the MOVIA Expert Working Group).

| **Country/region** | **Drugs available** | **Dosing** |
| --- | --- | --- |
| Hong Kong | CsA 0.05%, 0.1% and 0.5% | BID to QID |
| India | CsA 0.05% and 0.1%  Tacrolimus 0.03% | BID to QID  QD |
| Indonesia | CsA 0.05% and 0.1% | TID |
| Malaysia | CsA 0.05% | BID to QID |
| Singapore | CsA 0.05%, 0.1% and 0.5%  Tacrolimus 0.03% | BID to QID  QD |
| South Korea | CsA 0.02–0.1%  Tacrolimus 0.03–0.1% | QD to QID  QD |
| Taiwan | CsA 0.05% and 0.1% | QD to QID |
| Thailand | CsA 0.05% and 0.1%  Tacrolimus 0.03–0.1% | BID to QID  QD |
| Vietnam | CsA 0.05% | BID to QID |

*Tacrolimus use in patients with VKC may be off-label. The concentrations presented for tacrolimus and CsA include commercial and hospital-compounded formulas. Availability and access to treatments may vary across clinics, hospitals, regions, and countries. Each treatment option should be considered in accordance with the level of evidence available at the time of the decision.
BID: twice daily, CsA: cyclosporine A; N/A: not available; QD: once daily; QID: four times daily; TID: three times daily.
This table is correct as of January 2021.

**Supplementary Table 5.** Topical corticosteroids available for VKC by country (provided by the MOVIA Expert Working Group).

| **Country/region** | **Drugs available** | **Dosing** |
| --- | --- | --- |
| Hong Kong | Dexamethasone 0.1%  Fluorometholone 0.1%  Loteprednol 0.5%  Prednisolone acetate 1% | BID to QID  (as required) |
| India | Betamethasone 0.1%  Dexamethasone 0.1%  Loteprednol 0.5%  Fluorometholone 0.1%  Prednisolone acetate 1% | BID to QID  (as required) |
| Indonesia | Dexamethasone 0.1%  Fluorometholone 0.1%  Hydrocortisone 0.001–0.33%  Prednisolone acetate 1% | TID to QID  (as required) |
| Malaysia | Betamethasone 0.1%  Dexamethasone 0.1%  Loteprednol 0.5%  Fluorometholone 0.1%  Prednisolone acetate 1% | BID to QID  (as required) |
| Singapore | Betamethasone 0.1%  Dexamethasone 0.1%  Fluorometholone 0.1%  Loteprednol 0.5%  Prednisolone acetate 1% | QD to hourly  (as required) |
| South Korea | Dexamethasone 0.1–0.35%  Fluorometholone 0.1%  Loteprednol 0.5%  Prednisolone acetate 1%  Rimexolone 1% | QD to 12 times daily  (as required) |
| Taiwan | Betamethasone 0.1%  Dexamethasone 0.1%  Fluorometholone 0.1%  Loteprednol 0.5%  Prednisolone acetate 1% | QD to hourly  (as required) |
| Thailand | Dexamethasone 0.1%  Fluorometholone 0.1%  Loteprednol 0.2% and 0.5%  Methylprednisolone (hospital formulas)  Prednisolone acetate 1% | As required |
| Vietnam | Dexamethasone 0.1% (+ antibiotics; rarely used)  Fluorometholone 0.1%  Loteprednol 0.5%  Prednisolone acetate 1% | As required |

BID: twice daily, QD: once daily; QID: four times daily; TID: three times daily.
This table is correct as of January 2021.

**Supplementary Table 6.** Systemic pharmacological treatment available for VKC by country (provided by the MOVIA Expert Working Group).

| **Country/region** | **Drugs available** | **Dosing** |
| --- | --- | --- |
| Hong Kong | Cetirizine 10 mg  Desloratadine 5 mg  Fexofenadine 120 mg  Loratadine 10 mg | QD |
| India | Cetirizine 10 mg  Desloratadine 5 mg  Fexofenadine 120 mg  Loratadine 10 mg | QD |
| Indonesia | Cetirizine 10 mg | BID to TID |
| Malaysia | Cetirizine 10 mg  Desloratadine 5 mg  Fexofenadine 60 mg  Levocetirizine 5 mg  Loratadine 10 mg  Montelukast 4 mg, 5 mg and 10 mg | QD |
| Singapore | Cetirizine 10 mg  Desloratadine 5 mg  Fexofenadine 60 mg  Loratadine 10 mg  Pheniramine 25–150 mg  Terfenadine 60 mg and 120 mg | QD |
| South Korea | Cetirizine 10 mg  Desloratadine 5 mg  Fexofenadine 120 mg  Levocetirizine 5 mg  Montelukast 10 mg | QD |
| Taiwan | Cetirizine 10 mg  Desloratadine 5 mg  Fexofenadine 60 mg  Levocetirizine 5 mg  Loratadine 10 mg  Montelukast 5–10 mg | QD |
| Thailand | Cetirizine 10 mg  Desloratadine 5 mg  Fexofenadine 120 mg  Loratadine 10 mg | QD |
| Vietnam | Fexofenadine 120 mg  Loratadine 10 mg | QD |

BID: twice daily; QD: once daily; TID: three times daily.
This table is correct as of January 2021.
